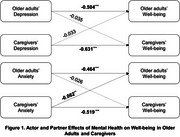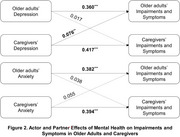# Relationship between mental health and health impairments in older adults and caregivers: A dyadic analysis

**DOI:** 10.1002/alz70860_103628

**Published:** 2025-12-23

**Authors:** Yeonju Jin, Timothy Reistetter, Ickpyo Hong

**Affiliations:** ^1^ Yonsei University, Wonju, Korea, Republic of (South); ^2^ UT Health San Antonio, San Antonio, TX, USA; ^3^ Yonsei University, Wonju, Gangwon‐do, Korea, Republic of (South)

## Abstract

**Background:**

Older adults and their caregivers often share a close, interdependent relationship due to caregiving activities, which can influence each other's physical and mental health. Caregiving imposes significant emotional and physical strain on caregivers, increasing their risk for anxiety, depression, and chronic health conditions. Similarly, older adults, who often face physical impairments and emotional challenges, are affected by the well‐being of their caregivers, creating a bidirectional dynamic. Understanding these reciprocal effects is important for designing interventions to improve health outcomes within caregiving dyads.

**Method:**

This retrospective, cross‐sectional study applied the Actor‐Partner Interdependence Model (APIM) from 971 dyads extracted from the 2022 National Health and Aging Trends Study (NHATS) and the National Survey of Caregiving (NSOC). As part of a dyadic analysis, structural equation modeling was used to estimate actor effects (effects on one's own outcomes) and partner effects (effects on a partner's outcomes). Depression and anxiety were used as independent variables, while dependent variables included total scores of six impairments and symptoms (e.g., pain) and ten well‐being items (e.g., feeling cheerful).

**Result:**

Older adults were mostly aged 75–79 years (21.6%), and caregivers averaged 62.8 years (SD = 14.7). Most caregivers were daughters (33.1%) or spouses/partners (30.1%). Caregivers were most commonly daughters (33.1%) or spouses/partners (30.1%). In APIM results, Caregivers' anxiety was negatively associated with older adults' well‐being (*β* = ‐0.062, SE = 0.028, *p* = 0.030), while older adults' anxiety showed no significant partner effect. Depression had no partner effects. Depression and anxiety were negatively associated with their own well‐being (*β* = ‐0.504 to ‐0.631, *p* < 0.001). Caregivers' depression was significantly associated with older adults' impairments and symptoms (*β* = 0.076, SE = 0.030, *p* = 0.011), while older adults' depression showed no significant partner effect. Anxiety showed no significant partner effects for either group. Depression and anxiety were positively associated with their own impairments and symptoms (*β* = 0.360 to 0.417, *p* < 0.001).

**Conclusion:**

This study emphasizes the interdependence within caregiving dyads and highlights the need to address their reciprocal dynamics when developing interventions to enhance health outcomes for both caregivers and care recipients.